# Fused Filament Fabricated Poly(lactic acid) Parts Reinforced with Short Carbon Fiber and Graphene Nanoparticles with Improved Tribological Properties

**DOI:** 10.3390/polym15112451

**Published:** 2023-05-25

**Authors:** Anzum Al Abir, Dipto Chakrabarti, Bruno Trindade

**Affiliations:** CEMMPRE—Centre for Mechanical Engineering, Materials and Processes, University of Coimbra, Rua Luís Reis Santos, 3030-788 Coimbra, Portugal

**Keywords:** PLA composite, 3D printing, mechanical and tribological characterization, short carbon fiber, graphene nanoplatelets

## Abstract

This study investigated the mechanical and tribological properties of 3D-printed Poly (lactic acid) (PLA) composites reinforced with different concentrations of carbon fibers (SCF) and graphene nanoparticles (GNP) (0.5 to 5 wt.% of each filler). The samples were produced using FFF (fused filament fabrication) 3D printing. The results showed a good dispersion of the fillers in the composites. SCF and GNP promoted the crystallization of the PLA filaments. The hardness, elastic modulus, and specific wear resistance grew with the increase in the filler concentration. A hardness improvement of about 30% was observed for the composite with 5 wt.% of SCF + 5 wt.% GNP (PSG-5) compared to PLA. The same trend was observed for the elastic modulus with an increase of 220%. All the composites presented lower coefficients of friction (0.49 to 0.6) than PLA (0.71). The composite PSG-5 sample showed the lowest value of specific wear rate (4.04 × 10^−4^ mm^3^/N.m), corresponding to about a five times reduction compared to PLA. Therefore, it was concluded that the addition of GNP and SCF to PLA made it possible to obtain composites with better mechanical and tribological behavior.

## 1. Introduction

3D printing additive manufacturing (AM) has allowed the reshaping of numerous crucial component manufacturing industries for aerospace, automotive, semiconductor, and biomedical applications [1].

There is a growing interest in developing high-performance polymer matrix composites suitable for 3D printing by adding different materials into the polymer matrix. Among these, short carbon fibers (SCF), carbon nanotubes (CNT), glass fibers, and graphene nanoparticles (GNP) are promising fillers to overcome the existing limitations of the polymer matrix by creating a composite with better structural and functional properties that none of the constituents alone could achieve [2,3]. Poly(lactic acid) (PLA) is a potential biodegradable polymer used as a matrix in polymer-based composites. PLA-graphene nanocomposite blends are actively being employed to produce 3D-printed scaffolds for tissue engineering [4]. Although the biocompatibility of this composite has been demonstrated in previous studies [5,6], its prospective application in load-bearing structures, as well as the resulting performance under varying loading situations, should be investigated further. When polymers are used in tribological applications at great velocity under high loads, they exhibit a low-load-carrying capacity and a short running life [7], with considerable financial loss to industry [8]. There is currently significant interest in applying polymer-based composites for tribological applications [9,10,11]. Carbon fillers, including carbon nanotubes (CNT), carbon fibers (CF), graphene, and others, can be used to improve the tribological behavior of polymer composites [1]. Because of their excellent thermal, mechanical, and electrical properties, high surface-to-volume ratio, and good dispersion in polymer matrices, SCF and GNP are good candidates for the advancement of structural and functional polymer composites [12,13].

Previous studies have indicated that incorporating carbon fiber can enhance the mechanical properties of printed PLA, with short and continuous carbon fibers positively affecting the tensile and shear modulus in various printing directions [14,15]. Further, continuous carbon fiber can reduce the failure strain of PLA, while increasing its tensile and flexural behavior [15]. Similarly, introducing graphene nanoplatelets into PLA can affect its mechanical properties depending on factors, such as raster direction and nanoplatelet size. While some studies have found that graphene-reinforced PLA may have poor mechanical properties, others have shown that its capabilities can be improved by adjusting the raster orientation or using larger nanoplatelets [16,17,18]. Multiwalled carbon nanotubes have also been studied as reinforcements for PLA, with concentrations exceeding 1 wt.% found to significantly enhance its mechanical properties [19,20]. In addition, the incorporation of multiple carbon nanofillers has been investigated due to their impact on the mechanical properties of PLA nanocomposites, with Batakliev et al. [21] showing that combining GNP and MWNCT (multi-wall carbon nanotubes), nanocomposites with better mechanical properties could be obtained, and Basheer et al. [22] demonstrated that adding SCF and graphene to PLA tripled its mechanical strength.

With respect to the tribological properties, there are few available studies on the effect of adding carbonaceous fillers to PLA. Batakliev [23] studied the influence of MWCNTs and GNPs on the tribological behavior of PLA using scratch and wear experiments, and claimed that the scratch resistance of the 12 wt% GNP/PLA nanocomposite was twice as high as neat PLA. Bustillos et al. [24] studied the influence of graphene on PLA and showed that PLA-graphene composites presented a significant improvement in creep and wear resistance. Suresha et al. [25] have demonstrated that adding 20 wt.% short carbon fibers to PLA resulted in composites with improved wear resistance (70% decrease in specific wear rate). In a recent study on PLA-based biocomposites reinforced with SCF, GNP, and SCF + GNP [26] produced by casting, we have shown that the joint addition of these two fillers (volume ratio between the PLA and the fillers of 1:0.02) had a beneficial effect on the hardness and specific wear rate of PLA.

Based on the available literature, two conclusions can be drawn. First, although the beneficial effect of adding carbonaceous fillers on the mechanical properties of 3D-printed PLA is well documented, their influence on tribological behavior needs to be studied further. Second, there are no studies concerning the combined effect of SCF and GNP on both the mechanical and tribological behavior of 3D-printed PLA. Therefore, the focus of this work was to combine the effect of the solid lubrication of graphene with the increase in mechanical strength induced by SCF to produce SCF/GNP-reinforced PLA 3D-printed parts using fused filament fabrication (FFF) with improved mechanical and tribological properties. FFF is currently widely used for the manufacture of thermoplastic parts, essentially due to the low cost of the equipment and the extensive variety of filaments available on the market. PLA granules were mixed with different concentrations of SCF and GNP (0.5 to 5 wt.% of each filler) and melted together at 200 °C. The maximum added content of reinforcements was intended not to drastically decrease the elastic strain of the composites and to allow adequate extrusion of the filaments. The mixtures were granulated and extruded to produce filaments. Finally, 3D parts were printed using FFF. The influence of the filler content on the structure, mechanical, and tribological properties is presented and discussed.

## 2. Materials and Methods

**Figure 1:**
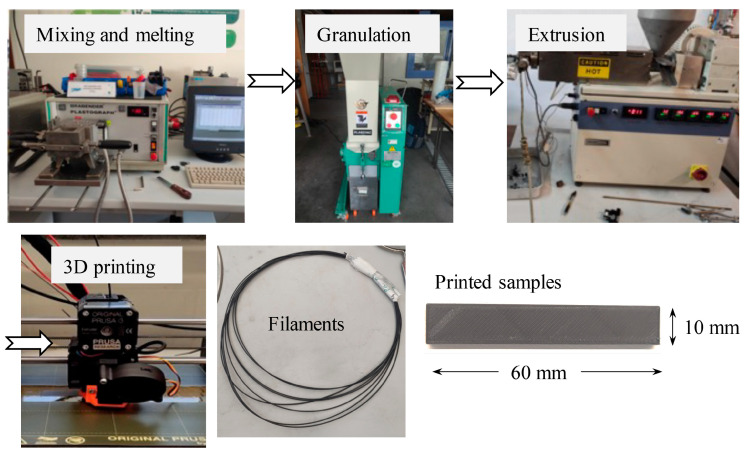
3D-printed samples’ manufacturing process: mixing and melting of the PLA granules with GNP and SCF, granulation of the mixtures, extrusion of the filaments, and 3D printing. The filaments and the 3D samples produced are also shown.

The composite samples produced in this work (Table 1) were fabricated in four stages: mixing and melting of the PLA granules with GNP and SCF, granulation of the mixtures, extrusion of the filaments, and 3D printing (Figure 1). The PLA granules (average diameter of 4.85 mm and density of 1.25 g/cm^3^) were supplied by Goodfellow (Huntingdon, UK). SCF (density of 1.8 g/cm^3^) was provided by Sigrafil (Wiesbaden, Germany) with an average filament length of 80 μm and a diameter of 7 μm. GNP (purity 99.9% and density of 2 g/cm^3^) with an average thickness of 5 nm and a length of 30 μm was supplied by Nanografi (Jena, Germany).

The PLA granules and the reinforcements were mixed and melted at 200 °C for 30 min using Brabender Plastograph equipment (Duisburg, Germany). The torque was continuously measured as a function of time during this process. The mixtures obtained were then granulated in Plaszone equipment (Moita, Portugal). After that, the filaments were produced by extrusion using Brabender equipment. The 3D-printed samples were produced using FFF (Prusa i3 MK3 3D printer, Prague, Czech Republic). The 3D printing parameters are listed in Table 2.

The samples printed were analyzed using different techniques. The morphological analysis was performed by means of a scanning electron microscope (SEM) (Hitachi-SU3800, Tokyo, Japan). Energy-dispersive X-ray spectroscopy (EDS) (X-MaxN, Oxford Instruments, Abingdon, UK) was used to determine the chemical composition. The crystalline structure was investigated using X-ray diffraction (XRD) (Philips XPert, Malvern Panalytical Ltd, Malvern, UK) with Co-Ka radiation (1.77889 Å). X-ray microtomography (SKYSCAN, Bruker microCT Systems, Bremen, Germany) was used to evaluate the quality of the filaments and 3D-printed samples. The thermophysical behavior and crystallinity of the PLA matrix were evaluated using differential scanning calorimetry (DSC) (NETZSCH-DSC 204 f1 Phoenix, Selb, Germany). The tests were performed from 25 °C to 230 °C with a heating rate of 10 °C/min and a flux of 40 mL/min of N_2_. The crystallinity of the PLA was evaluated using the following equation [20]:

(1) $$Xc\;\left(\%\right)=\left(\frac{∆H_{f}}{∆H_{f0}}\right)\times 100$$

where Δ*H_f_* and Δ*H*_*f*0_ represent the melting enthalpy and the standard enthalpy of PLA (93 J/g [27]), respectively.

The hardness of the samples was determined using Shore D hardness (CV Instruments Limited, Sheffield, UK). In this test, a needle is placed on the top of the sample and pressure is applied. The resistance to penetration is rated on the scale. The ASTM D2240-00 standard was used. Five tests were performed on each sample in different areas of the flat top surface.

The impulse excitation technique was used to calculate the elastic modulus. Five tests were performed on each sample according to the standard ASTM C1259-14. The elastic modulus was calculated using Equation (2):

(2) $$E=0.9465\left(\frac{mft^{2}}{d}\right)\left(\frac{l^{3}}{t^{3}}\right)T_{1}$$

where l, t, and d are the dimensions of the sample (length = 60 mm, width = 10 mm, and thickness = 3 mm, respectively), m is the mass and ft is the fundamental frequency of the first flexural vibration mode. Because of the finite dimensions of the samples, a correction factor $T_{1}$ is needed. For its calculation, a constant Poisson ratio of 0.3 was assumed.

The tribological properties were determined in dry conditions by means of ball-on-disk tests and a reciprocating mode (Rtec instrument-MFT 5000, Yverdon-les-Bains, Switzerland). The tests were performed with a load of 5 N on ultrasonically cleaned (ethanol) stainless steel (100 Cr6) balls with a diameter of 5 mm. The tests were performed for 300 s, with a stroke length of 6 mm, and a frequency of 8.5 Hz. The experiments were carried out at 25 °C with 50 % humidity. The wear tracks and scars on the samples were analyzed using 3D profilometry (Alicona-InfiniteFocus, Bruker Alicona, Leicestershire, UK).

The specific wear rate, *K*, was calculated using Equation (3) [28]:

(3) $$K=\frac{V}{NS}\;$$

where V is the wear volume on the sample, N is the load applied, and S is the distance of the journey.

## 3. Results and Discussions

### 3.1. Raw Materials

The characterization of the raw materials was carried out in a previous study and published in [26]. The PLA granules were mostly spherical (equivalent to a diameter of approximately 4.8 mm), with 45% of crystallinity. They exhibited a glass temperature (*Tg*) of 60 °C and a melting temperature (Tm) of 173 °C. The GNP powder presented a flake-like morphology with stacked layers of graphite sheets, and the SCF consisted of cylindrical rods with a length of tens of micrometers and diameters lower than 10 μm.

### 3.2. Mixture of PLA with the Reinforcements

**Figure 2:**
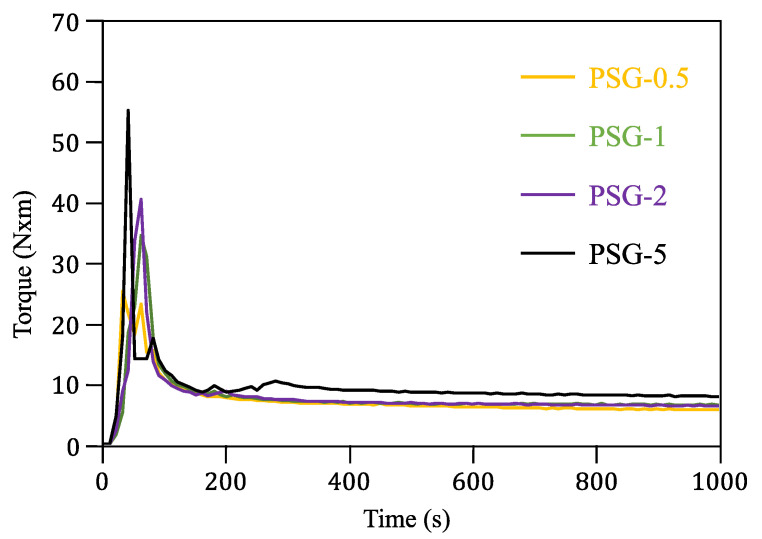
Torque vs. time curves of the reinforced PLA samples.

The torque vs. time curves recorded during the mixture of the raw materials is presented in Figure 2. The maximum torque values occurred in the first seconds of the mixing process and depended on the composition of the samples, i.e., the higher the concentration of SCF and GNP, the higher the maximum torque. This can be explained by the plasticity of the raw materials. Both fillers are ceramic materials with higher hardness and lower ductility than PLA. After 200 s, all the curves became stable and horizontal meaning that the mixtures were homogenous after that time.

### 3.3. Production of the Filaments

**Figure 3:**
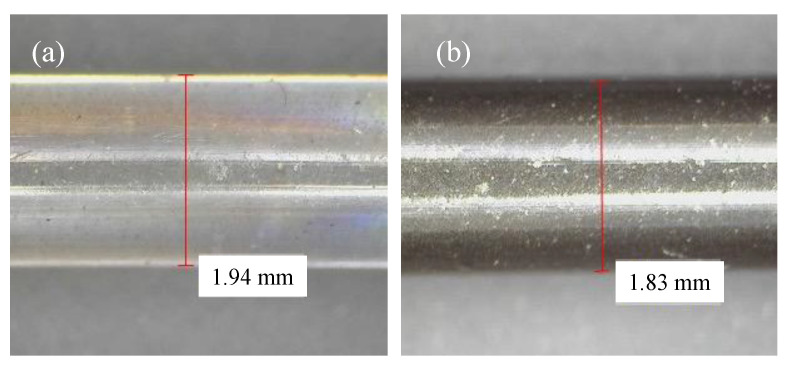
Optical images of the (**a**) PLA and (**b**) PSG-5 filaments.

The mixtures were extruded to produce the filaments and subsequently inspected using optical microscopy. Figure 3a,b shows optical images of the PLA and PSG-5 filaments, respectively. They presented some variations in diameter (from 1.75 to 1.94 mm). The average diameter of the filaments was 1.8 mm, suitable for 3D printing. The parts of the filaments with a diameter greater than 1.85 mm were not used to print the samples, in order to avoid nozzle-clogging issues, and to maintain the uniformity of the samples printed.

**Figure 4:**
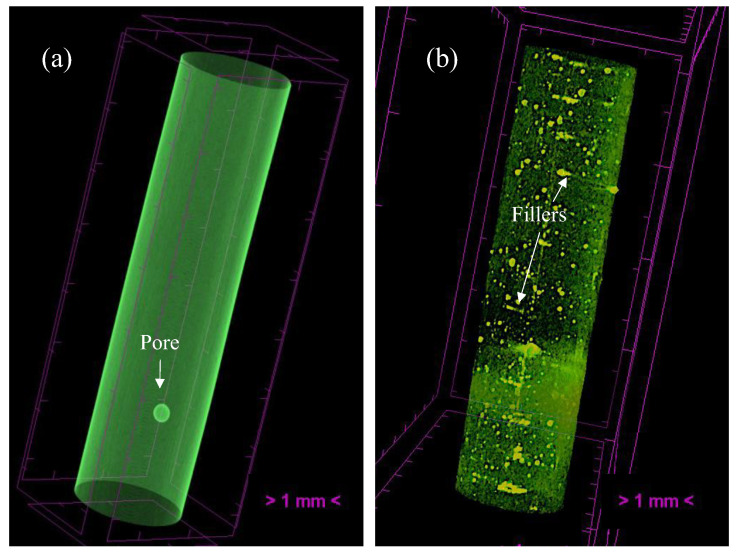
Microtomography images of (**a**) PLA and (**b**) PSG-5 filaments, as typical examples of all the other filaments.

The tomography results of the extruded PLA and PSG-5 filaments are illustrated in Figure 4a,b, respectively. The filaments were quite dense. The GNP and SCF reinforcements (yellow dots—Figure 4b) were homogeneously distributed throughout the filaments (Figure 4b).

**Figure 5:**
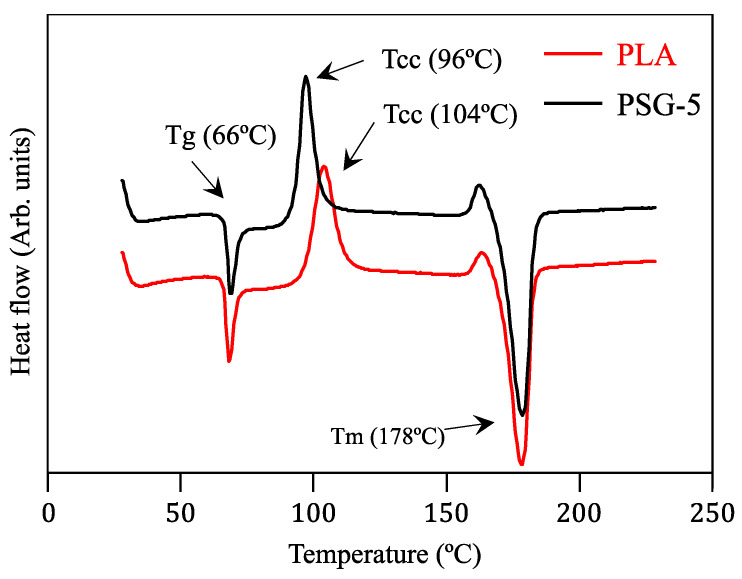
DSC curves of the PLA and PSG-5 filaments.

The DSC curves of these filaments are illustrated in Figure 5. The curves are identical and characterized by three peaks corresponding to the glass transition (Tg), cold crystallization (Tcc), and melting (Tm) temperatures. The only difference concerned the cold crystallization temperature. The reinforced PLA sample (PSG-5) showed a Tcc value 8 °C lower than that of PLA, which suggests that SCF and GNP acted as nucleating agents and lowered the crystallization temperature. Similar results were obtained by Ruz-Cruz et al. [29] on PLA-based multiscale cellulosic biocomposites, and Vinyas et al. [30] on PLA + 10% carbon fibers. The existence of Tcc in both DSC curves means that the partial amorphization of PLA took place during the processing of the filaments since this peak was not observed for raw PLA [16]. Analogous results were reported by Sorrentino et al. [31].

#### D-Printed Samples

**Figure 6:**
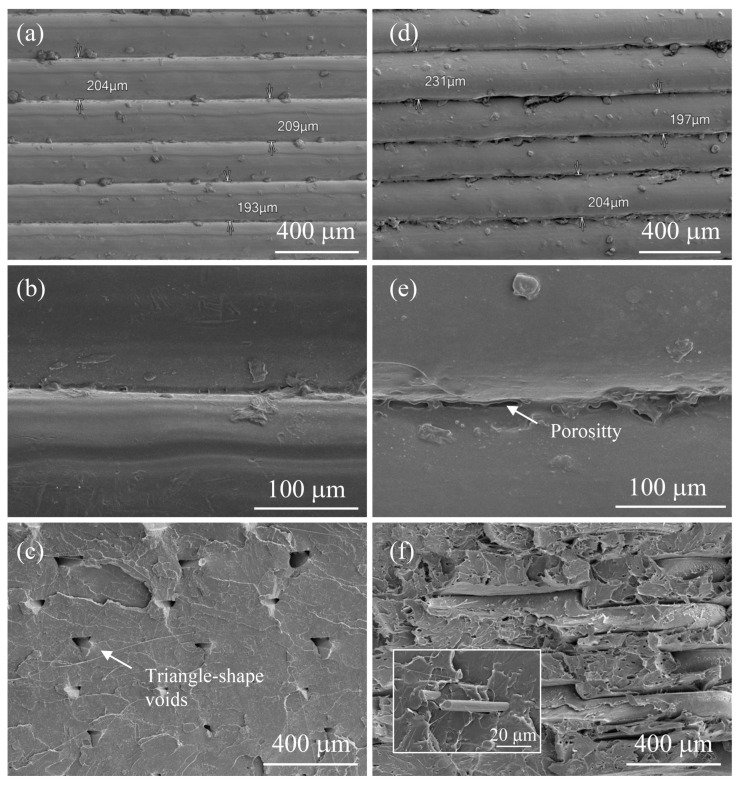
SEM images of (**a**–**c**) PLA and (**d**–**f**) PGS-5 samples printed. (**a**,**b**) Cross-section view, (**b**,**e**) the interface between layers, and (**c**,**f**) the fracture surface. The inset in Figure (**f**) shows a carbon fiber in the PLA matrix.

Figure 6a–f shows SEM images of the PLA and PSG-5-printed samples to serve as typical examples of all the others. The average thickness of the layers was 200 μm (Figure 6a,c). A higher porosity was observed at the interface of the composite samples (Figure 6b,d). The images of the surfaces fractured in liquid nitrogen (Figure 6c,e) revealed a more ductile behaviour of the PLA when compared to the composite samples and the partial separation of the various layers printed in the case of the composite samples. This is related to the fragile behavior of SCF and likely a poor wettability between the PLA matrix and fillers. Triangle-shape voids, which were formed during printing [14,32], were observed on the fracture surface of the PLA samples.

**Figure 7:**
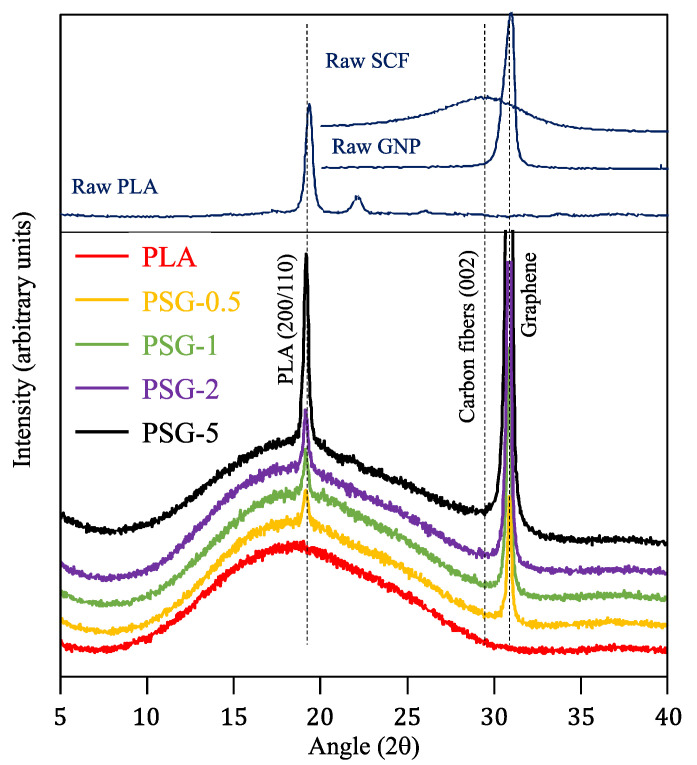
XRD patterns of the samples printed.

**Figure 8:**
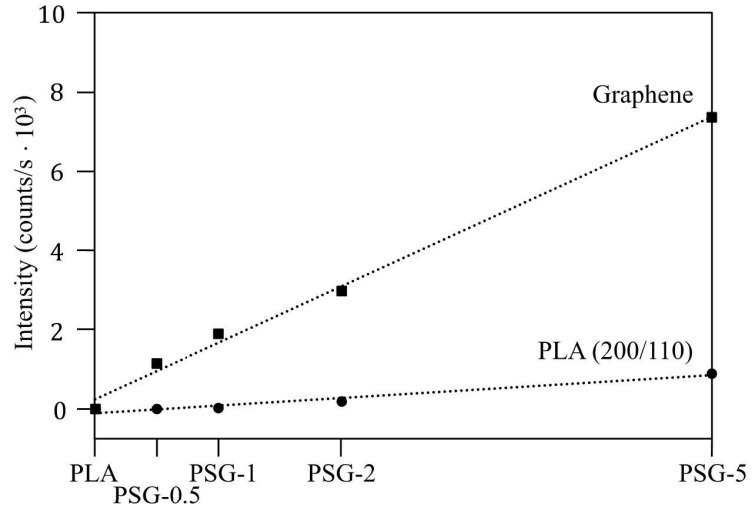
The intensity of PLA (200/110) and graphene diffraction peaks of the samples printed.

The XRD patterns of the 3D-printed PLA and PGS-5 samples are shown in Figure 7. The PLA presented just one broad peak centered at 20°, corresponding to an amorphous structure. During the 3D printing process, the rapid cooling rate impeded the crystallization of PLA. Besides this peak, the reinforced PLA samples also showed diffraction peaks corresponding to the (200/110) plane of PLA (2θ = 19.1°) and graphene (2θ = 30.9°). The intensity of both peaks showed a direct dependence on the concentration of the filler (Figure 8), meaning that, as mentioned before, SCF and GNP accelerated the crystallization of PLA. Similar results were observed in different studies where other reinforcements were added to PLA [29,30]. The XRD peak of SCF was not detected due to its low intensity and overlapping with the amorphous peak of PLA.

**Figure 9:**
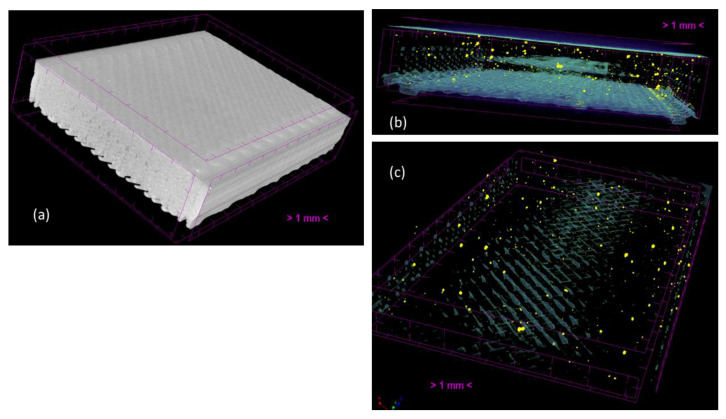
Microtomography images of (**a**) PLA, (**b**) PSG-5 (front view) and (**c**) PSG-5 (isometric view) samples printed.

The microtomography images of the PLA and PSG-5 samples printed are illustrated in Figure 9. The 0.2 mm layer-by-layer construction is clearly noticeable (Figure 9a). The 45° raster angle is visible on the surface of the samples (Figure 9a,c). The dispersed GNP and SCF particles (yellow dots) were all over the sample printed (Figure 9b,c).

Table 3 shows the hardness (H), elastic modulus (E), and the H/E ratio of the 3D-printed samples. The H/E ratio represents the elastic strain to failure, which is strongly correlated with energy dissipation in mechanical contact. Usually, this relationship is calculated considering the unit of GPa for both properties, H and E. This is not the case in this work as the hardness was evaluated using the Shore D hardness test. Consequently, the values should not be compared with the ones from the literature and serve only to establish a relationship between the various samples produced.

Concerning hardness, the values measured varied from 63.4 to 81.1 Shore D. No significant differences in the hardness were detected in the different top surface areas tested, with standard deviation values being quite low.

PLA presented the lowest value (63 Shore D). The reinforced samples showed higher hardness with PSG-5 having the highest value (81 Shore D). The PSG-1 and PSG-2 samples had the same average hardness (79 Shore D). The hardness of a material depends on its chemical, physical, and mechanical properties. In addition, porosity also contributes to the variation of this property. No significant porosity variation was observed with the addition of carbon fillers. Therefore, the addition of GNP and SCF to PLA was responsible for the increase in hardness. It is reported in the literature that even a small amount of GNP and SCF can boost the hardness of polymer-based composites [3,33,34,35]. This happens because both fillers are harder than PLA.

Regarding the elastic modulus, the values increased as the concentration of the fillers in the PLA matrix increased. The PLA presented an elastic modulus of 1.86 GPa, a value slightly higher than the one reported by Leite et al. [36]. However, the values are lower than the typical elastic modulus of commercial PLA obtained using traditional processes (3.5 GPa) [37,38] due to the low crystallinity and the high number of pores of the 3D-printed PLA. As observed for hardness, the addition of just 0.5 wt.% of SCF and 0.5 wt.% of GNP significantly increased the elastic modulus (2.35 GPa). Samples with 1 wt.% and 2 wt.% of both fillers showed similar elastic moduli of 3.14 GPa (PSG-1) and 3.28 GPa (PSG-2). The highest value (4.11 GPa) was obtained for the PSG-5 sample (highest concentration of fillers). These results are in accordance with previous studies on PLA-based composites reinforced with GNP or SCF [1,27,39,40,41,42]. These reinforcements have a higher tensile strength and elastic modulus than PLA, which restrict the movement of the polymeric chains, leading to improved resistance to strain and an increased load-bearing capacity. As a result, the reinforced PLA samples are able to resist the loads applied more effectively than only PLA.

As expected, the H/E ratios of the samples printed showed a decrease with the increase in the concentration of fillers. As mentioned before, the addition of GNP and SCF to PLA increased both H and E. However, its influence on E was predominant, and, therefore, the elastic strain to breakage is decreased by increasing the content of fillers in PLA.

**Figure 10:**
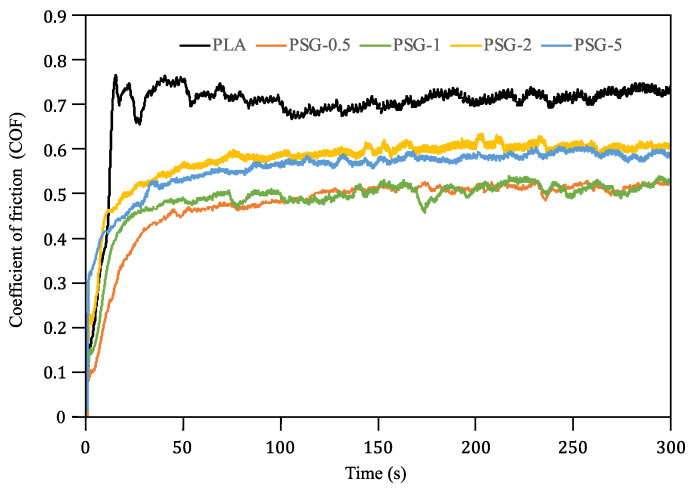
CoF curves of the 3D-printed samples.

The CoF curves of the 3D-printed samples are presented in Figure 10. Small amounts of SCF and GNP (0.5 wt%) were enough to significantly reduce the CoF. All the composite samples showed lower CoF values (0.49 to 0.6) compared to PLA (0.71). The PSG-0.5 and PSG-1 samples had the lowest value (~0.49) among all the composite samples. The further increase in SCF and GNP contents (2 wt.% and 5 wt%) led to an increase in CoF (0.6). These results are in tune with those reported by Hanon et al. [42], although the polymer was different (polyurethane-based resin) in that study and only graphene was used as the reinforcement. During the reciprocating ball-on-disk tests, the carbon flakes provided a transfer of the film that acted as a solid lubricant [43], reducing the CoF of PLA [44]. The addition of high concentrations of fillers (mainly SCF) gave rise to harder and less flexible composites. As the fibers do not bend as easily in contact with the counter body, this led to more friction. Moreover, SCF presented more surface asperities, which may also cause more friction.

**Figure 11:**
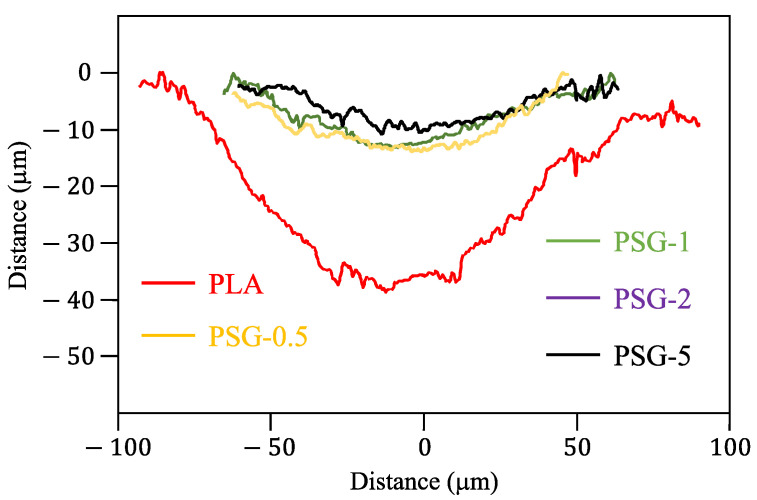
Wear profiles of the 3D-printed samples.

**Figure 12:**
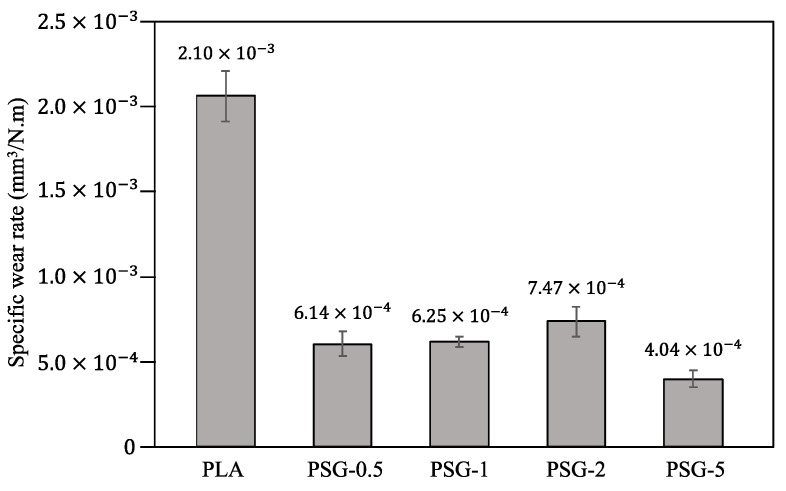
Specific wear rates of the 3D-printed samples.

Figure 11 shows the 3D profilometry images of the printed samples’ wear profiles. The specific wear rates are illustrated in Figure 12. All the composite samples presented lower wear depths than the PLA. The PSG-5 sample showed the lowest value (11 µm), much lower than PLA (50 µm). The PSG-0.5 and PSG-1 samples had similar values, close to 15–16 µm. The PSG-2 sample had the highest maximum wear depth (18 µm) among them.

PLA presented a specific wear rate of 2.1 × 10^−3^ mm^3^/N.m. The incorporation of SCF + GNP into PLA led to a decreased wear rate. The lowest value was obtained for the PSG-5 sample (4.04 × 10^−4^ mm^3^/N.m) corresponding to about a five times reduction compared to PLA. All the other composite samples showed lower specific wear rates (from 6.14 × 10^−3^ to 7.47 × 10^−3^ mm^3^/N.m) than PLA but higher than PSG-5. Similar results were obtained by Bustillos et al. [24] for GNP-reinforced PLA. Friedrich [45] also demonstrated that SCF can significantly improve the wear resistance of PEEK + PTFE-engineered plastics. Both the SCF and GNP are harder than PLA, which increases the composites’ hardness and wear resistance. Additionally, SCF increases the region of contact surface with higher load-bearing capacity, providing these composites with higher wear resistance. During the sliding wear tests, there is a high probability that these particles will be released by the composites and roll between the sliding surfaces. Subsequently, they act as solid lubricants, minimizing the contact points between the counter bodies and the surface of the composites, which leads to lowered CoF and wear [43,46]. Moreover, the SCF and GNP fillers reduce the concentration of stress during the wear tests, and the formation of a network of microcracks, resulting in a dramatic decrease in wear [47]. The carbon fibers act as a reinforcement phase, capable of distributing the load over a larger area. This helps to reduce the concentration of stress at the surface and increases the material’s wear resistance. Additionally, the carbon fibers act as a barrier to prevent the polymer matrix from deforming, which also improves the material’s wear resistance. With increasing carbon content, the hardness and elastic modulus of the composites also increase, which means they are able to withstand large loads without breaking or deforming. As a result, the overall improvements in the composite material’s mechanical properties help to increase the wear resistance.

**Figure 13:**
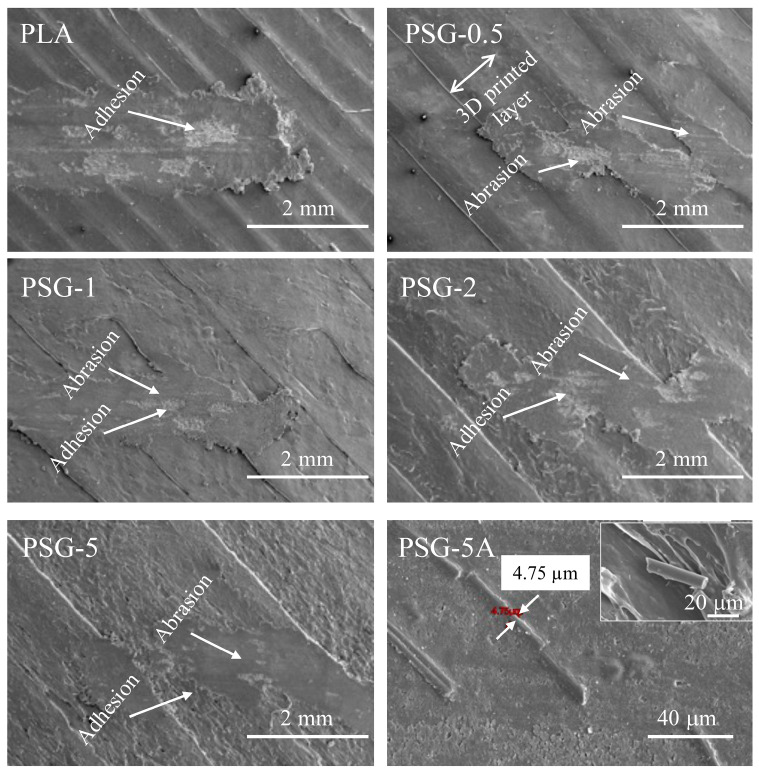
SEM images of the wear scars on the 3D-printed samples after the tribological tests.

The wear mechanisms on the worn surfaces were characterized using SEM (Figure 13). The worn surface of the PLA mostly demonstrated the adhesive wear mechanism that led to wear debris emerging as a result of an adhesion process caused by plastic deformation and shear. This conclusion is consistent with Lancaster’s work [48].

Abrasion, along with adhesion, was the most common wear process in all the reinforced PLA composites. SCF particles emerged on top of the composite surfaces during the sliding of the steel counter body (Figure 13—PSG-5A). The wear of the composites was mainly due to a three-body abrasive wear mechanism. A similar phenomenon has already been documented in other studies [24,41]. The SCF assisted in supporting the applied load and protected the PLA matrix from wear. GNP acted as a solid lubricant, reducing the direct contact between the steel balls and the PLA matrix; thus, preventing easy removal of the polymeric matrix. This explains why the wear was significantly lower in the composites compared to PLA.

Finally, one may say that the increase in the fillers content (from 0.5 to 5 wt.%) was responsible for the increase in the mechanical properties and load-bearing capacity, and the decrease in the plastic deformation, which led to the enhanced specific wear resistance of the composites. Although samples of PLA reinforced with only one kind of mono filler were not produced in this work, it is possible to state that the results are in line with a previous study on the fabrication of PLA bio-composites by mechanical alloying and casting [26]. That is, there was a combined effect of the solid lubrication of graphene and the increase in hardness and elastic modulus achieved using SCF.

## 4. Conclusions

The results of this study allowed us to draw the following conclusions: The manufacturing process used made it possible to obtain filaments, with an appropriate distribution of SCF and GNP. These fillers led to an increase in the hardness and elastic modulus of PLA and contributed to solid lubrication. The mechanical and tribological properties of the samples produced showed a direct dependence on the filler content. The PSG-5 sample (5 wt.% of SCF and 5 wt.% of GNP) showed a 30% increase in hardness compared to unreinforced PLA (81.1 and 33.4 Shore D, respectively) and a 220% increase in modulus of elasticity (4.11 and 1.86 GPa, respectively). Regarding the tribological behavior, the reinforced PLA samples showed lower friction coefficients than the non-reinforced PLA as well as a decrease in the specific wear rate. The PSG-5 sample had the lowest value (4.04 × 10^−4^ mm^3^/N.m) corresponding to about a five times reduction compared to PLA. Finally, the PLA-based composites produced in this work are promising materials for biomedical applications, e.g., the manufacturing of improved PLA-based scaffolds.

## Figures and Tables

**Table 1:** Name and chemical composition of the samples produced in this work.

Sample Name	PLA (wt.%)	SCF (wt.%)	GNP (wt.%)
PSG-0.5	99	0.5	0.5
PSG-1	98	1	1
PSG-2	96	2	2
PSG-5	90	5	5

**Table 2:** 3D printing parameters.

Parameter Name	Value
Printed sample dimensions (mm)	60 × 10 × 3
Extruder Temperature (°C)	215
Bed	Painter’s tape
Bed Temperature (°C)	60
Cooling (Fan speed)	100%
Layer Thickness (mm)	0.2
Raster Angle (°)	−45/+45
Infill Density (%)	100%
Nozzle diameter (mm)	0.8

**Table 3:** Hardness (H), elastic modulus (E), and H/E ratio of the 3D-printed samples.

Sample	H (Shore D)	E (GPa)	H/E
PLA	63.4 ± 0.8	1.86 ± 0.01	33.9
PSG-0.5	77.8 ± 1.0	2.35 ± 0.01	33.2
PSG-1	79.4 ± 0.5	3.14 ± 0.01	25.2
PSG-2	79.2 ± 0.7	3.28 ± 0.02	24.1
PSG-5	81.1 ± 1.0	4.11 ± 0.04	19.7

## Data Availability

Not applicable.
